# Conjoint analysis of clinical, imaging, and pathological features of schistosomiasis and colorectal cancer

**DOI:** 10.3389/pore.2023.1611396

**Published:** 2023-11-30

**Authors:** Fang Zhang, XiaoShuang Wang, YuanTing Zhu, Peng Xia

**Affiliations:** ^1^ Department of Radiology, Jingzhou Hospital Affiliated to Yangtze University, Jingzhou, China; ^2^ Department of Pathology, Jingzhou Hospital Affiliated to Yangtze University, Jingzhou, China; ^3^ Department of Parasitology, School of Basic Medicine, Health Science Center, Yangtze University, Jingzhou, China

**Keywords:** schistosomiasis, colorectal cancer, imaging, t stage, pathology

## Abstract

This study aims to examine and compare clinical, radiological, and pathological data between colorectal cancer (CRC) patients with and without schistosomiasis and uncover distinctive CRC characteristics when accompanied by schistosomiasis. This retrospective study is based on data collected from 341 patients diagnosed with CRC post-surgery and pathology. Of these patients, 101 (Group A) were diagnosed with colorectal cancer co-occurring with schistosomiasis (CRC-S), while 240 patients (Group B) were diagnosed with colorectal cancer without concurrent schistosomiasis (CRC-NS). Both groups were compared and analyzed based on their clinical data, imaging-based TNM staging, lymph node metastasis, nerve invasion, vascular cancer thrombus, and histopathological differentiation. A Chi-squared test revealed a significant difference in gender distribution between the patients with CRC-S (Group A) and CRC-NS (Group B), with a *p* -value of 0.043 and χ^2^ = 4.115. Specifically, a higher incidence rate was observed among males in Group A. There was a difference in the overall distribution of TNM staging between the two groups (*p* = 0.034, χ^2^ = 6.764). After pairwise comparison, a statistically significant difference was observed in the T3 stage (*p* <0.05). The proportion of the T3 stage in Group A was significantly higher than that in Group B, indicating certain advantages. There was a difference in postoperative histopathological grading between the two groups (*p* = 0.005, χ^2^ = 10.626). After pairwise comparison, a statistically significant difference was observed between the well-differentiated adenocarcinoma and the moderately and poorly differentiated adenocarcinoma (*p* <0.05), with a higher proportion of welldifferentiated patients in Group A compared to Group B. There was no significant difference in age, lymph node metastasis, nerve invasion, and vascular invasion between the two groups of patients (*p* > 0.05). Among the 101 patients with CRC-S, 87 (86%) showed linear calcification on CT imaging. Patients with CRC-S are mainly male, with tumor staging mostly in the middle stage, high tumor differentiation, and low malignancy. CT imaging can help identify the presence of lumps and linear calcification indicative of schistosome deposits. MRI can early clarify TNM staging and determine the presence of lymph node metastasis and nerve and vascular invasion.

## Introduction

CRC ranks third among the most prevalent cancers worldwide, constituting 8.5% of all cancer mortalities [1]. The underlying causes of CRC remain undefined; however, epidemiological and pathological evidence proposes its association with schistosomiasis [2–4]. However, a series of chronic intestinal diseases caused by schistosomiases, such as intestinal fibrosis and ulcers, are considered precancerous lesions [5]. Compared with CRC-NS, CRC-S has different clinical and pathological features, which are complex. Therefore, it is necessary to diagnose CRC-S accurately. The clinical history of schistosomiasis infection, elevated serum CA-125 levels, and imaging examinations have important guiding significance for diagnosing CRC-S. Currently, surgical treatment is considered one of the most effective treatment methods for CRC. Imaging can accurately carry out TNM staging of CRC and has the characteristics of multi-directional and high-resolution imaging. In terms of morphology, it can clearly show the related anatomical structure of CRC and reflect the characteristics of molecular diffusion and blood flow perfusion of pathological water in terms of function. It plays a critical role in the selection and evaluation of preoperative methods of clinical surgery, as well as the formulation of postoperative radiotherapy and chemotherapy treatment plans and the evaluation of efficacy. This study comprehensively analyzes the characteristics of CRC-S disease from clinical and imaging aspects combined with postoperative pathology.

## Materials and methods

### Patients

From January 2020 to December 2022, a total of 341 cases of colorectal cancer diagnosed pathologically were collected from our hospital. Out of these, 101 cases (group A), including 73 males and 28 females, aged 45–88 years, were diagnosed with colorectal cancer complicated with schistosomiasis by pathological examination after the operation. The remaining 240 patients had colorectal cancer without schistosomiasis (group B), including 144 males and 96 females, aged 35–82 years. Regardless of whether neoadjuvant radiotherapy and chemotherapy were performed before surgery, both groups of patients should undergo CT and 3.0T high-resolution MRI before surgery and neoadjuvant radiotherapy and chemotherapy. The Ethics Committee of the Faculty of Medicine of Yangtze University reviewed and approved this study (ethical code: KY202320), and the informed consent forms of tissue specimens, medical history, and data were signed.

### Imaging examination

Routine scanning was carried out using the Philips Brilliance CT 64 Slice scanner and the GE Discovery MR 750 3.0T MRI. The abdominal CT employed a plain scan and enhancement, with scan/reconstruction layer thickness set at 5.0 mm and 1.0 mm, respectively, and a scanning interval of 0.7 mm. All images were subsequently transmitted to the workstation for further evaluation. The MRI sequences employed included FSE axial T1WI, axial T2WI, sagittal T2WI, coronal T2WI, and DWI. All sequences covered the entire tumor, and the images were subsequently forwarded to the ADW4.6 workstation for post-processing to observe the tumor lesion’s location, size, and TNM staging.

### Data analysis

The TNM staging for CRC in this study adheres to the criteria provided by the American Joint Commission on Cancer (AJCC)/Union for International Cancer Control (UICC)/TNM staging system for CRC, as per the eighth edition in 2017: T1 stage tumors invade the mucosa and submucosa, T2 stage tumors invade the intrinsic muscle layer, T3 stage tumors penetrate the intrinsic muscle layer to the subserosal layer, or invade colorectal tissue without peritoneal coverage, T4 stage tumors penetrate the peritoneal visceral layer, or directly invade or adhere to other organs or structures. The histopathological grading for CRC follows the WHO 2010 edition: High differentiation implies >95% glandular duct formation; Middle differentiation: 50%–95% glandular duct formation; Low differentiation: 0%–49% glandular duct formation. Two highly experienced physicians, each with years of expertise in abdominal and pelvic imaging diagnosis, conducted a joint review and analysis of the tumor’s location, CT and MRI imaging characteristics, and its potential invasion of surrounding intestinal structures. If there was any disagreement, a consensus was reached through discussion. All cases underwent surgical and pathological diagnosis, and the staging, pathological tissue grading, presence or absence of lymph node metastasis, nerve invasion, and vascular cancer thrombus of the rectal tumor were recorded.

### Statistics

The statistical software SPSS 26.0 was used to compare various parameters, including age, gender, imaging TNM staging, presence or absence of lymph node metastasis, nerve and vascular invasion, and histopathological grading between patients in Group A (CRC-S) and Group B (CRC-NS). The collected data underwent subsequent analysis and processing. We employed the Chi-squared (χ^2^) test to compare usage rates, with a statistically significant result set at *p* < 0.05.

## Results

**FIGURE 1:**
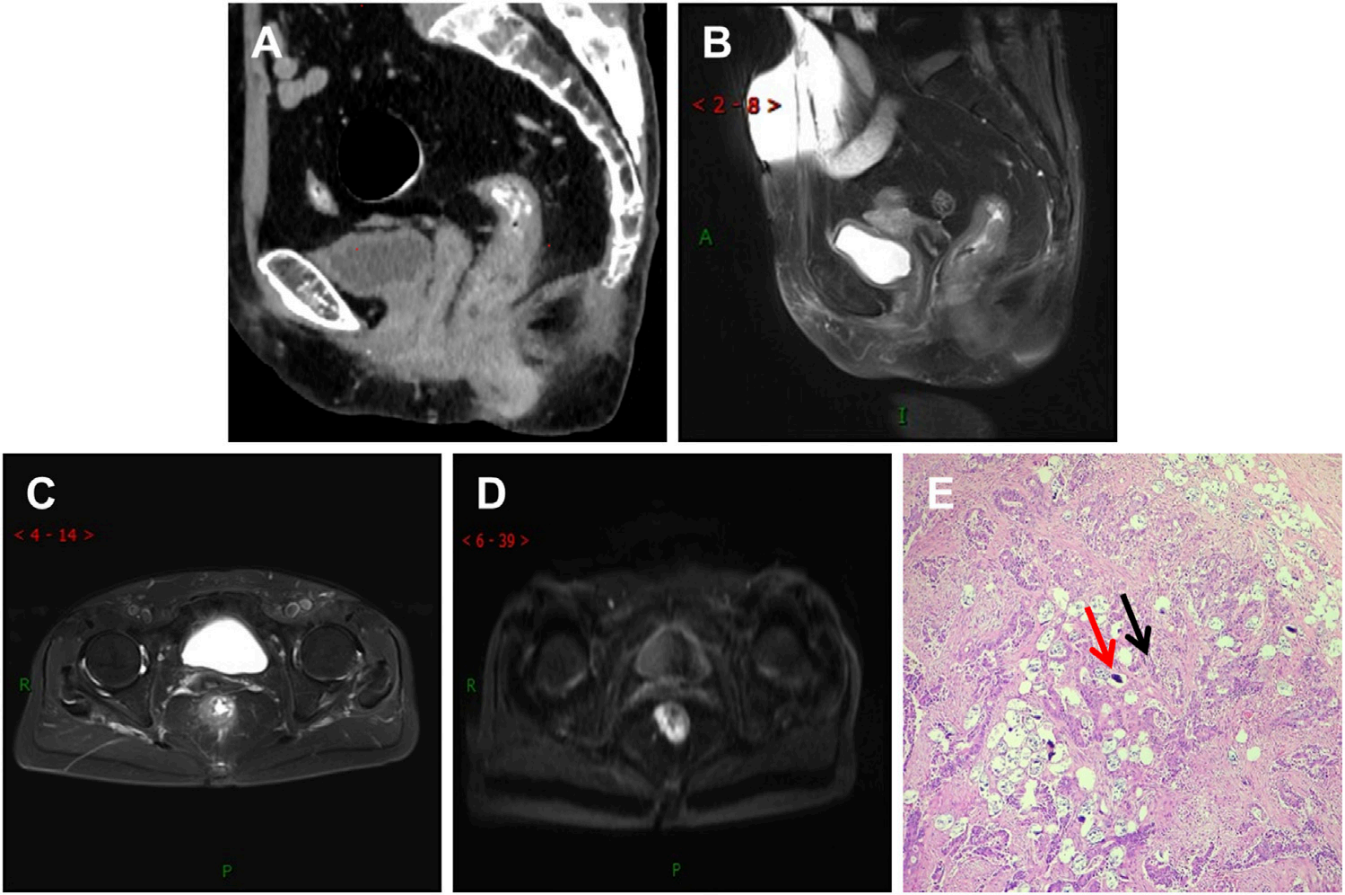
Differentiated adenocarcinoma of the rectum, where the tumor penetrates the intrinsic muscle layer to the exterior of the intestinal wall (T3N0MX stage). **(A)** CT shows significant thickening of the lower rectal wall and narrowing of the lumen, with multiple spotted and small strip-shaped calcifications visible within it; **(B,C)** T2WI sagittal and transverse images showed that the rectal tumor presented low signal intensity, thickening of the intestinal wall, rough edges, burry changes, incomplete muscularis mucosa lining, involvement of more than ½ circle of the intestinal cavity, and no lymph node shadow in the surrounding Mesentery fat space area; **(D)** The DWI sequence showed significantly high signal intensity in tumors; **(E)** In the pathological picture, the red arrow indicates Schistosoma eggs and the black arrow indicates tumor tissue. Schistosoma eggs are distributed in the tumor tissue.

**FIGURE 2:**
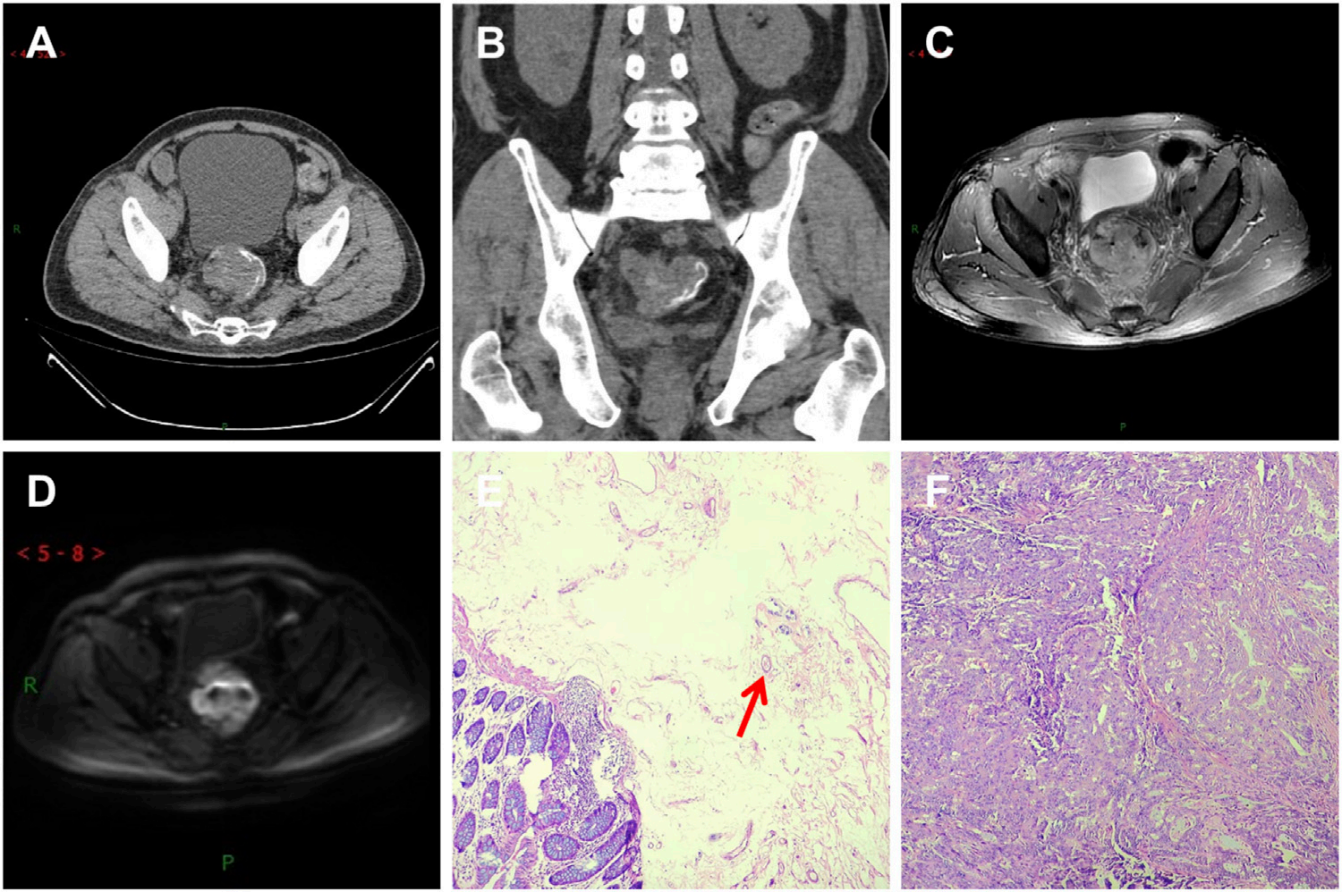
A patient with moderately differentiated adenocarcinoma of the rectum, with the tumor invading the entire intestinal wall and lymph node metastasis visible outside the intestinal wall (T3N1MX stage). **(A)** CT shows soft tissue mass shadows in the rectal area, linear calcification shadows in the submucosa of the intestinal wall, and lymph node shadows in the surrounding area; **(B,C)** T2WI transverse and sagittal images showed that the lesion involved all rectal walls and broke through the muscular layer. Burr-like and flocculent shadows were seen around the lesion. The boundary between the lesion and surrounding Mesentery fat was unclear. Slightly high signal lymph node shadows were seen in the rectal mesentery fat; **(D)** The DWI sequence showed significantly high signal intensity in the tumor; **(E)** In the pathological picture, the red arrow indicates the Schistosoma egg; **(F)** Tumor tissue.

This study compared Group A and Group B based on age, gender, imaging TNM staging, lymph node metastasis, nerve and vascular invasion, and histopathological grading. Group A showed significant differences (*p* < 0.05) compared to Group B in terms of gender, imaging TNM staging, and histopathological grading, with statistical significance (*p* < 0.05) (Table 1). There was a difference in the overall distribution of TNM staging between the two groups (*p* = 0.034, χ^2^ = 6.764). After pairwise comparison, a statistically significant difference was observed in the T3 stage (*p* < 0.05). The proportion of the T3 stage in Group A was significantly higher than that in Group B, indicating certain advantages. There was a difference in postoperative histopathological grading between the two groups (*p* = 0.005, χ^2^ = 10.626). After pairwise comparison, a statistically significant difference was observed between the well-differentiated adenocarcinoma and the moderately and poorly differentiated adenocarcinoma (*p* < 0.05), and the proportion of well-differentiated patients in group A was higher than that in group B (Table 1). CT showed thickening of the lower rectal duct wall with significant linear calcification indicative of Schistosoma (Figure 1A), MRI images clearly showed T3 staging (Figures 1B–D), and pathological images (Figure 1E) showed deposition of Schistosoma eggs within the tumor. In some cases, CT shows a clear mass with calcification in the rectal area (Figures 2A, B), while MRI images clearly show that the tumor has broken through the muscular layer in the T3 phase. The intestinal wall appeared significantly thickened with irregular masses, and there was evidence of infiltration into the fat spaces around the rectum and lymph node metastasis (Figures 2C, D). Pathological images (Figures 2E, F) further confirmed the presence of Schistosoma eggs and tumor tissue.

**TABLE 1:** Comparison of clinical, imaging, and pathological data between two groups of patients.

Parameter	Colorectal cancer		χ^2^	*p*-value
	Group A	Group B		
All cases	101	240		
Age (≤50/>50)	8/93	28/212	0.697	0.404
gender (male/female)	73/28	144/96	4.115	0.043
Imaging TNM staging (T1-2/T3/T4)	22/73/6	66/141/33	6.764	0.034
Imaging TNM staging	22/6	66/33	1.454	0.228
T1-2/T4				
Imaging TNM staging	22/73	66/141	2.401	0.121
T1-2/T3				
Imaging TNM staging	73/6	141/33	5.387	0.020
T3/T4				
Histologic grade (high/medium/low)	23/71/7	23/196/21	10.626	0.005
Histologic grade high/medium	23/71	23/196	10.232	0.001
Histologic grade high/low	23/7	23/21	4.513	0.034
Histologic grade medium/low	71/7	196/21	0.033	0.856
LN metastasis (Yes/No)	38/63	87/153	0.0138	0.907
Peripheral invasion (Yes/No)	30/71	62/178	0.362	0.548
Vascular invasion (Yes/No)	20/81	52/188	0.0576	0.810

## Discussion

Schistosomiasis has been classified as a clear Carcinogen for humans (Class I Carcinogen), and Schistosoma japonicum is considered a possible Carcinogen for humans (Class 2B Carcinogen) [6]. Schistosoma japonicum is the most prevalent in China, mainly concentrated in the middle and lower reaches of the Yangtze River. In recent years, significant progress has been made in controlling schistosomiasis, owing to advancements in national sanitary conditions and effective management. Studies have shown that [7, 8] because Schistosoma eggs deposit in the intestinal submucosa, the local inflammatory reaction forms Granuloma, and long-term repeated stimulation eventually leads to tissue proliferation, fibrosis, inflammatory polyps, and even the adenoma formation, which is consistent with the evolution process of colorectal tumors. Therefore, the inflammatory response caused by schistosomiasis plays an important role in cancer development. Various studies [9, 10] have affirmed schistosomiasis as an autonomous etiological factor for CRC. Current research endeavors have pivoted towards understanding the molecular dynamics associated with CRC-S. The prevalence of CRC-S is low, leading to a dearth of public awareness about routine health check-ups. Consequently, this lack of awareness contributes to significant delays in the initial diagnosis of CRC-S.

Findings from the current investigation reveal a male predominance in CRC-S cases, corroborating several domestic and international studies [11–13]. This trend is potentially linked to the increased likelihood of males encountering contaminated farmland water [14, 15]. Another research study [16] postulates an association with female hormone secretion, such as estrogen and progesterone. It has been reported [17, 18] that CRC-S mainly occurs in the rectum, followed by the Sigmoid colon, which is consistent with the results of this study. Age discrepancies among CRC-S and CRC-NS patients have varied in the literature; some suggest that CRC-NS patients tend to be older by 6–16 years compared to their CRC-S counterparts [15, 19], whereas others propose that CRC-S patients are typically older [20]. However, no substantial age differences were observed between the two cohorts in our study.

Surgical treatment of CRC is the primary clinical approach and also serves as a critical adjuvant therapy for advanced rectal cancer stages T3 and beyond. It holds the potential to prognosticate patient outcomes effectively. Routine check-ups, colonoscopies, and barium enemas can establish an initial CRC diagnosis without staging. Endoscopic ultrasound enables detailed visualization of local tumor invasion, yet its scope is restricted. If a tumor results in lumen constriction, the endoscope’s passage becomes hindered, precluding examination. Submucosal dissection via endoscopy has gained traction as a common early-stage CRC treatment, although it cannot detect deeper lesions, lymph node metastasis in the intestinal periphery, or vascular invasion. Hence, MRI has emerged as the benchmark imaging modality for evaluating CRC [21].

Out of the 101 CRC-S patients in this retrospective analysis, 87 (86%) exhibited linear calcification on CT imaging, 92 (91%) displayed marked intestinal wall thickening, and 9 (9%) had pronounced masses. Zhang et al. [22] compared CT findings with pathological attributes, identifying irregular intestinal wall thickening in 95% of patients and soft tissue masses in 5%. Among these patients, 80% or 104 presented with linear, patchy, and minor calcifications, while unclear margins were observed in 96 out of 130 patients. The study by Hao Feng et al. [23] aligns with our findings, as their 26 patients demonstrated CT-detected intestinal wall thickening accompanied by linear calcification.

This study used a 3.0T high-resolution MRI sequence to carry out T staging of CRC, including the degree of intestinal wall thickening, stenosis, peritubular invasion, mesorectal fascia invasion, lymph node metastasis, vascular tumor thrombus, and nerve invasion. T staging indicates the primary tumor’s growth range and the degree of its invasion into surrounding tissues. For CRC visualized via MRI, distinguishing between stages T2 and T3 proves crucial and challenging for preoperative clinical staging. A determining factor is whether the tumor breaches the serous layer of the mesorectum, that is, the completeness of the low signal ring within the rectal muscle layer, clarity of surrounding fat spaces, and involvement of the mesorectum. Occasionally, the boundary between the rectal tumor edge and the muscularis can appear indistinct and irregular, forming low signal, needle-like, or spiny extensions in the surrounding area, often misconstrued as tumor invasion of the rectal mesothelium. However, it may be a response reflecting proliferative connective tissue in the rectal tumor’s surrounding mesothelium, devoid of tumor cell invasion [24]. Various factors, such as the depth of tumor infiltration, lymph node metastasis, and differentiation degree, directly impact the prognosis of rectal cancer.

Schistosoma has little effect in the early stage of T1-2 in different clinical stages, while patients with T3-4 stage tumors complicated with lymph node metastasis have a certain risk in the late clinical stage. There was a difference in the overall distribution of TNM staging between the two groups (*p* = 0.034, χ^2^ = 6.764). After pairwise comparison, a statistically significant difference was observed in the T3 stage (*p* < 0.05). The proportion of the T3 stage in Group A was significantly higher than that in Group B, thus indicating certain advantages. This is roughly similar to the findings of the previously reported study [12]. Another previous study [14] has indicated an older average age among schistosomiasis patients, with most CRC-S patients found in stages T1-3, predominantly in the early and middle stages, which is largely consistent with this study. In these cases, clinicians initially assess the presence of calcification, intestinal thickening, or mass of Schistosoma japonicum using CT scans. Following this, a high-resolution MRI can be employed to determine whether the rectal tumor has invaded the muscularis, assess the integrity of the muscularis, and identify whether the tumor has broken through the muscularis and the depth of infiltration, identify the presence of cancerous nodules in the surrounding mesorectum, and ascertain if there is an invasion of the mesorectal fascia and peritoneum. In general, total mesorectal excision (TME) is often used for direct resection in T2-stage cases. Patients with advanced stage T3 and above mainly require neoadjuvant radiotherapy and chemotherapy before surgery. This aims to reduce the staging, enhance the rate of complete tumor resection, and reduce the potential risk of recurrence, which might otherwise impact the rate of anus-preserving procedures. Accurately distinguishing T2/3 colorectal cancer helps determine whether patients need preoperative radiotherapy and chemotherapy. For patients who do not need these treatments, it helps reduce the complications associated with them, lessen the economic burden on patients, and save treatment time. For patients who need preoperative radiotherapy and chemotherapy, TME after neoadjuvant radiotherapy and chemotherapy can significantly improve the tumor resection rate and reduce the risk of postoperative recurrence. The similarity between imaging and pathological results implies that imaging examinations for comprehensive analysis, evaluation, and prognosis of CRC-S patients preoperatively can significantly enhance clinical diagnostic efficacy, reduce patient treatment duration, and provide compelling evidence for preoperative treatment plans.

Previous research suggests [25] that CRC-S exhibits a lower risk of lymph node metastasis, which may be associated with the development of submucosal fibrous tissue in the intestinal wall, forming a restrictive band that impedes the metastasis of cancer cells *via* lymphatic channels. Another explanation could be the presence of Schistosoma egg granulomas in tissue vessels and lymphatic nodes, which may obstruct or damage their structure, resulting in intestinal wall fibrosis and lymphatic vessel occlusion, thereby making tumor metastasis rare. However, other studies [11, 12] report a higher predisposition toward lymph node metastasis in CRC-S. The results of this investigation indicate that the likelihood of lymph node metastasis in CRC-S is not significantly different from that in CRC-NS, possibly due to the stage, number, and clinical pathological changes of the CRC-S cases examined. No significant differences were observed between the two patient groups concerning lymph node metastasis, neural invasion, and vascular cancer thrombosis, suggesting that a schistosomiasis infection may not elevate the risk of neural and vascular invasion in CRC. There was a difference in postoperative histopathological grading between the two groups (*p* = 0.005, χ^2^ = 10.626). After pairwise comparison, a statistically significant difference was observed between the well-differentiated adenocarcinoma and the moderately and poorly differentiated adenocarcinoma (*p* < 0.05), and the proportion of well-differentiated patients in group A was higher than that in group B. This suggests that the degree of differentiation in CRC-S is higher, potentially leading to a better prognosis.

Schistosoma japonicum infection is considered an important risk factor for CRC and can increase the mortality of CRC patients [17], but the exact pathogenesis is still unclear. It is widely believed that chronic inflammatory processes and oxidative stress are the reasons for the carcinogenic effect of schistosomiasis infection [26]. Research reports using the AOM/DSS model demonstrate that the protein Sj E16.7 secreted by Schistosoma eggs can promote cancer progression [27]. In addition to immune response, various other factors, such as oncogene activation, tumor suppressor gene inhibition, changes in normal microRNA expression patterns, genetic factors, and gut microbiota, are all involved in developing CRC [28]. Multivariate analysis revealed that schistosomiasis impacted cancer patients’ disease-free and overall survival rates. It has been suggested that the prognosis for CRC-S is poorer than that for CRC [29]. Conversely, another study ^14^ reported a significantly higher 5 years survival rate in CRC-S, compared to the CRC-NS group, as determined through follow-up surveys. While there is inconsistency in the literature regarding the prognosis for these two groups, the author posits that this discrepancy may be due to factors such as the study cases being drawn from differing periods and alterations in various pathological processes. Therefore, irrespective of the prognosis, early accurate diagnosis, staging, and treatment planning are critical for patients with CRC-S.

There are certain limitations to this study. Due to the retrospective analysis, there may be bias in selecting patients. However, schistosomiasis does have adverse effects on CRC patients, and a large amount of data has not yet been collected using methods such as CT and MRI for preoperative staging of rectal cancer and their comparison. Further improvement is needed in future research.

In summary, compared to CRC-NS, CRC-S is more common in males and predominantly at the mid-stages as assessed by imaging and pathology. The level of pathological differentiation tends to be high, and the risk of lymph node metastasis is not increased. CT imaging and high-resolution MRI serve as critical tools in the accurate and early staging of patients with CRC-S. These techniques are integral in prognostic evaluation before surgery, including assessing the tumor size, potential invasion of surrounding mesorectal fascia, lymph node metastasis, vascular tumor thrombosis, and nerve invasion. Such insights guide clinical surgeons in selecting surgical procedures and rationally applying postoperative treatment plans.

## Data Availability

The original contributions presented in the study are included in the article/supplementary material, further inquiries can be directed to the corresponding author.
